# The Loudness Dependence of Auditory Evoked Potentials (LDAEP) as an Indicator of Serotonergic Dysfunction in Patients with Predominant Schizophrenic Negative Symptoms

**DOI:** 10.1371/journal.pone.0068650

**Published:** 2013-07-12

**Authors:** Christine Wyss, Konrad Hitz, Michael P. Hengartner, Anastasia Theodoridou, Caitriona Obermann, Idun Uhl, Patrik Roser, Edna Grünblatt, Erich Seifritz, Georg Juckel, Wolfram Kawohl

**Affiliations:** 1 Department of Psychiatry, Psychotherapy and Psychosomatics, Psychiatric University Hospital Zurich, Zurich, Switzerland; 2 Department of Psychiatry, Ruhr University Bochum, LWL University Hospital, Bochum, Germany; 3 Department of Child and Adolescent Psychiatry, University of Zurich, Zurich, Switzerland; Baylor College of Medicine, United States of America

## Abstract

Besides the influence of dopaminergic neurotransmission on negative symptoms in schizophrenia, there is evidence that alterations of serotonin (5-HT) system functioning also play a crucial role in the pathophysiology of these disabling symptoms. From post mortem and genetic studies on patients with negative symptoms a 5-HT dysfunction is documented. In addition atypical neuroleptics and some antidepressants improve negative symptoms via serotonergic action. So far no research has been done to directly clarify the association between the serotonergic functioning and the extent of negative symptoms. Therefore, we examined the status of brain 5-HT level in negative symptoms in schizophrenia by means of the loudness dependence of auditory evoked potentials (LDAEP). The LDAEP provides a well established and non-invasive in vivo marker of the central 5-HT activity. We investigated 13 patients with schizophrenia with predominant negative symptoms treated with atypical neuroleptics and 13 healthy age and gender matched controls with a 32-channel EEG. The LDAEP of the N1/P2 component was evaluated by dipole source analysis and single electrode estimation at Cz. Psychopathological parameters, nicotine use and medication were assessed to control for additional influencing factors. Schizophrenic patients showed significantly higher LDAEP in both hemispheres than controls. Furthermore, the LDAEP in the right hemisphere in patients was related to higher scores in scales assessing negative symptoms. A relationship with positive symptoms was not found. These data might suggest a diminished central serotonergic neurotransmission in patients with predominant negative symptoms.

## Introduction

Negative symptoms are core features of schizophrenia and are generally considered in psychiatric scales [1] and diagnostic classification, e.g. in the DSM-IV-TR [2]. These symptoms describe a deficit or an absence of normal mental functions and have traditionally been considered to consist of affective flattening, alogia, avolition, anhedonia and attentional impairment [3]. Research in this field has characterised negative symptoms to occur as accompanying symptoms of positive symptoms (e.g. hallucinations, delusion and formal thought disturbances) and both in the prodromal and residual state of the disease. They are named primary negative symptoms if directly related to the disease process itself and not resulted in a secondary action from other psychiatric symptoms or medication side effects [4]. Negative symptoms often lead to social impairment, resulting in poor success in social and professional life and account for much of the long-term morbidity and poor functional outcome of patients with schizophrenia [5], [6]. Despite that, the pathophysiology of negative symptoms has been widely unknown so far.

Schizophrenia research on biochemical functions has largely focused on the dopamine neurotransmitter system. The dopamine-hypothesis based on imaging studies proposes an imbalance of cortical and subcortical preponderance of dopaminergic neurotransmission, where a subcortical hyperstimulation of dopamine D2-receptors leads to positive symptoms, and a hypoactivation of cortical dopamine D1-receptors leads to negative and cognitive symptoms [7], [8], [9], [10]. However, the theory of a serotonin (5-hydroxytrypamine, 5-HT) and dopamine interaction as the mechanism behind schizophrenia has gained more acceptance. Moreover, there is evidence that the serotonin system inhibits dopamine function in frontal cortex and reinforce the imbalance in the mesolimbic-mesocortical pathway of the dopaminergic system [11], [12], [13], [14], [15]. The involvement of the serotonergic system in this theory is due to the fact that atypical neuroleptics [16], [17], [18] and antidepressants [19], [20], [21], which act via the serotonergic system, show remarkable potency for the treatment of negative symptoms. Meltzer [22] specifies that 5-HT2A receptor antagonism and 5-HT1A partial agonism together with weak dopamine D2 receptor antagonism are responsible for the principal pharmacologic effects of atypical neuroleptics on negative symptoms. An addendum to the former concept was that this new hypothesis allows explaining the heterogeneity of schizophrenia even better. Since a single type of abnormality of the neurotransmitter systems is unlikely to emerge as characteristic of all patients with schizophrenia. To sum up, there is evidence that the serotonergic system is a key component in the pathogenesis of negative symptoms.

The serotonin system plays an important role in pathophysiology of the major psychiatric disorders and provides a target of pharmacotherapeutic interventions. Therefore reliable indicators of this system are in urgent need for clinical and scientific interest [23]. Such indicators could be used after overcoming some challenges concerning the implementation in daily clinical use to identify patients with serotonergic dysfunctions and thus serve as therapy predictors [24], [25]. In fact, common indicators of the serotonin system are mainly indirect peripheral parameters that only give an approximate indication of the central serotonergic system. Such methods as neuroendocrinological challenge tests, measuring concentrations of serotonin metabolites in cerebrospinal fluid and tryptophan depletion test have not been proven to be sufficiently valid [26]. Furthermore, the use of imaging techniques that allow to reflect the availability of binding potentials of serotonin transporter (SERT) or 5-HT receptors, such as positron emission tomography (PET) [27] and single photon emission computed tomography (SPECT), are not appropriate for daily clinical use because of their invasive properties [15].

In the continuing search for biological correlates of psychiatric disorders, evoked potentials now constitute a prime target of investigation. In particular, the loudness dependence of auditory evoked potentials (LDAEP) has been widely reported to be a valid measure of central serotonergic activity in humans [25], [28], [29], [30], [31], [32]. This measure represents a growth of the amplitude in primary auditory cortices, measured from the peak of the N1 to the peak of the P2 component along with an increase in sound pressure level (Figure `). A pronounced LDAEP supposedly reflects a low central serotonergic neurotransmission and vice versa. Some other reports have suggested that the interpretation may be more complex and the LDAEP’s specificity as a marker of serotonin function has been challenged [33], [34], [35], [36].

A significant body of research documents a weaker LDAEP in patients with schizophrenia compared to healthy controls, thus indicating increased serotonergic activity in patients [37], [38], [39], [40]. But this research to date has tended to focus on the diagnosis of schizophrenia, neglecting the clinical heterogeneity. However, it is of great interest to investigate schizophrenia on the psychopathological symptom level. Thus, the aim of the current study is to scrutinize the putative role of serotonergic neurotransmission of negative symptoms in schizophrenia.

## Methods

### Subjects

The sample included 26 male subjects (13 patients, 13 controls) who underwent electrophysiological recording. Subjects with psychiatric comorbidity, drug or alcohol abuse, benzodiazepine consumption for more than 10 days before examination or a lifetime history of neurological diseases were excluded. Thirteen patients with predominant negative symptoms recruited from the Department of General and Social Psychiatry at the Psychiatric University Hospital Zurich met the diagnostic criteria for chronic paranoid schizophrenia in accordance to ICD-10 [41]. The psychopathological state of all patients was rated based on the Positive and Negative Syndrome Scale (PANSS; [42]) and the Scale for Assessment of Negative Symptoms (SANS; [1]). To differentiate depressive symptoms from negative symptoms, Hamilton Depression Rating Scale (HAMD; [43]), Bech-Rafaelsen Melancholia Scale (BRMES; [44]) and Calgary Depression Rating Scale for Schizophrenia (CDSS-G; [45]) were applied. All patients were using atypical antipsychotics during the test period. Dosages were transformed into chlorpromazine (CPZ) equivalent values for comparative reasons [46]. Thirteen healthy, age- and gender-matched volunteers recruited from medical staff and students served as the control group. Controls with a lifetime history of any psychiatric disorder were excluded.

### Ethics Statement

The study was approved by the special subcommission for psychiatry of the ethics committee of the canton of Zurich (“SPUK ZH Psychiatrie”) under the title “Die Rolle der zentralen serotonergen Aktivität bei Negativsymptomen” (Ref.-Nr.: E-19/2006) and was carried out in accordance with the Declaration of Helsinki. All subjects have given written informed consent. Only participants with uncompromised capacity to consent were approached. The capacity to give consent had been established by the senior consultant psychiatrists responsible for the treatment of the patients.

### Electrophysiological Assessment

Subjects were seated with their eyes open in a quiet room adjacent to the recording apparatus and were asked to avoid facial muscle movements throughout the auditory stimulus presentation sequence and the recording. As attention to the auditory stimuli has been shown to modulate the auditory evoked potentials [47] and therefore also the LDAEP [48], a silent movie was shown to them for distraction and the stimuli were presented in randomized orders and points in time that precluded preparatory state. Auditory evoked potential (AEP)-recording was performed with 32 electrodes referenced to FCz (BrainCap-MR 32 standard, 32 channels, Easycap, Herrsching-Breitbrunn) in accordance with the international 10–20 System [49]. Scalp electrode impedances were kept below 10 kΩ. Sinus tones (1 000 Hz, 40 ms duration with 10 ms rise and fall time, ISI randomized between 1 800 and 2 240 ms) of five intensities (60, 70, 80, 90, 100 dB sound pressure level, generated by a PC-stimulator) were presented binaurally in a pseudo-randomized order over headphones using Presentation software (Neurobehavioral Systems, Inc. San Pablo, CA). Data were collected with a sampling rate of 250 Hz and a band pass filter (0.5–70 Hz). Continuous EEG files for each subject were loaded into Brain Electrical Source Analysis software (BESA, version 5.3, MEGIS, Gräfelfing, Germany) and filtered digitally with a high bandpassfilter of 0.16–30 Hz (6/12 dB octave). Before averaging, the first responses of each of the five intensities were excluded in order to reduce short-term habituation effects. For artefact suppression, all trials were automatically excluded from averaging when the voltage exceeded ±50 µV in any of the 32 channels at any point during the averaging period. Data with a 100 ms pre stimulus and a 300 ms post stimulus baseline interval were then inspected visually. On average 63% (±5.228) artefact-free sweeps per intensity were averaged separately for each participant, which should lead to an appropriate signal-to-noise ratio.

### Dipole Source Analysis (DSA) and Single Electrode Estimation

Dipole source localization of the N1/P2-component of AEPs was computed by means of the inverse solution as implemented in BESA, using a spherical head model. DSA provides an important methodological advance, because overlapping subcomponents of the N1/P2-component in the primary as well as secondary auditory cortex can be studied separately [50]. This is a pivotal point, as primary auditory cortex is highly innervated by serotonin compared to secondary auditory cortex [51]. Similar studies reveal a high spatio-temporal accuracy with DSA [52], [53]. Based on the grand average over all subjects a dipole model was computed for the 60 dB and 70 dB intensities with two regional sources (one for each hemisphere). Several authors suppose a frontal protective mechanism being activated during presentation of high tone intensities [54], [55]. Therefore a third regional source was added to the frontal region for the high intensity dipole model computed for the 90 and 100 dB intensities. These two models were applied to the individual data sets (high intensity model to 60–70 dB, low intensity model to 80–100 dB) in order to obtain the spatio-temporal information of the brain activation. The methods have been published in detail elsewhere [28], [29], [51].

Because the majority of studies on the LDAEP focused on the N1/P2 component, which seems to be more internally consistent and test-retest reliable than slopes based on other components [56], [57], the peak-to-peak N1/P2 amplitudes were used to quantify differences in the responses to the different tone intensities. Additionally to the DSA approach we analysed the data with a scalp method, as recommended by our group [58], to facilitate across-study comparisons. N1/P2 amplitudes were determined at the Cz electrode and were re-referenced to linked mastoids. The LDAEP was determined by the median of all slopes of each possible connection between the five different N1/P2 amplitudes corresponding to the five different intensities [29] for tangential dipole activity of both hemispheres and Cz-electrode estimation derived amplitudes. These values were used as the main variables for statistical evaluation.

### Statistical Analysis

Comparison of age and smoking status in patients and controls was conducted with a t-test for independent samples and cross-tabulation with χ^2^ test, respectively. To test the association between LDAEP values and the group factor (control group vs. schizophrenic patients) we conducted a series of generalized linear models (GLM) [59]. GLM was chosen because it allows for variables that are not normally distributed in comparison to familiar used methods as ANOVA or linear regression analysis. LDAEP of the left and right hemisphere and from Cz-estimation were entered as the dependent variables. The covariates age and nicotine use were tested separately in bivariate analyses against LDAEP using DSA. Distribution and link-function of the LDAEP variables were chosen according to their graph and the goodness of model fit indices. For this purpose we compared the Akaike’s information criterion (AIC) and the Bayesian information criterion (BIC) for the different distributions and link-functions. The best fit to the data was finally obtained with a gamma distribution (right skewed distribution) and log link-function. In all GLM a robust estimator was used to reduce the effects of outliers and influential observations. Group effects on LDAEP were displayed with mean differences, whereas associations between continuous measures and LDAEP were depicted with unstandardized regression coefficients (B). In order to provide comparability among predictors all continuous covariates were standardized using the z-transformation. Wilcoxon-test was used to test if the medians between left and right LDAEP differed significantly. Analyses were carried out with SPSS version 20 for Windows.

## Results

Demographics and psychopathology data for both groups are summarised in Table 1. Although antipsychotic medication estimated by CPZ-equivalent dose had a medium to strong effect on psychopathological scales, the correlations did not reach the level of statistical significance (PANSS general score; r = −0.698, p = 0.08; other scales r = −0.31–0.44, p>0.1).

**Table 1:** Demographic and clinical data of the sample.

	Patients	Controls	t/χ2	p
N	13	13		
Age (years)	35.0 (8.13)	35.4 (8.17)	t = 0.120, df = 24	0.905
Medication (CPZ)	707.0 (597.62)	–	–	–
Smoking (yes/no)	69; 31	23; 77	?2 = 5.571, df = 1	0.018*
PANSS positive	15.46 (4.93)	–	–	–
PANSS negative	18.39 (6.25)	–	–	–
SANS composite score	31.31 (15.39)	–	–	–
BRMS	6.31 (3.77)	–	–	–
HAMD 17	8.69 (4.07)	–	–	–
CDSS-G	3.15 (3.11)	–	–	–

Data presented as % or mean ± SD. Abbreviations: CPZ, Chlorpromazine Dose Equivalence Ratios; PANSS, Positive and Negative Syndrome Scale; SANS, Scale for Assessment of Negative Symptoms; BRMS, Bech-Rafaelsen Melancholia Scale; HAMD, Hamilton Depression Rating Scale, CDSS-G, Calgary Depression Rating Scale for Schizophrenia.

* p<0.05.

The LDAEP using DSA was significantly associated with the group membership in both hemispheres (right: Wald = 10.094, df = 1, p = 0.001; left: Wald = 7.791, df = 1, p = 0.005). Patients with schizophrenia showed a significantly higher LDAEP than the control group (Table 2, Figure S2). Results were adjusted for age and nicotine use. The magnitude of the group effect on LDAEP on both hemispheres was remarkably large, as indicated through the standardized mean difference Cohen’s d = 1.04 (left) and d = 1.20 (right) (benchmarks are as follows: d = 0.3 depicts a small effect, d = 0.5 a medium effect and d = 0.8 a large effect). No significant differences in the LDAEP between the groups were found using single electrode estimation at Cz (Wald = 0.057, df = 1, p = 0.811). No significant differences between left and right LDAEP were found, neither among the whole sample (Z = −1.283, p = 0.200), nor among schizophrenic patients (Z = −1.153, p = 0.249) or the control group (Z = −0.524, p = 0.600).

**Table 2:** LDAEP mean values in left and right hemisphere and Cz electrode across groups.

Hemisphere	Group	Mean	95% CI	Wald χ^2^ (df)	Sig
Left	Controls	1.060	0.894–1.258	7.791 (1)	0.005*
	Patients	1.450	1.230–1.710		
Right	Controls	0.905	0.781–1.050	10.094 (1)	0.001*
	Patients	1.234	1.073–1.420		
Cz	Controls	0.150	0.116–0.194	0.057 (1)	0.811
	Patients	0.142	0.105–0.192		

Results are adjusted for age and nicotine use.

* p<0.01.

Moreover, we observed a significant positive relationship between the SANS subscales “affective flattening” (beta = 0.207, p = 0.000), “anhedonia” (beta = 0.155, p = 0.016) and “attentional impairment” (beta = 0.189, p = 0.015) and the LDAEP in the right hemisphere in patients. SANS composite score (the sum of scores for all items), which reflects severity of negative symptoms, was also positively correlated with the right LDAEP (beta = 0.153, p = 0.035) (Table 3). Depressive symptoms (BRMS and CDSS G scale) (beta = −0.372, p = 0.000; beta = −0.305, p = 0.000) as well as PANSS general score (beta = −0.159, p = 0.026) were associated with the left LDAEP. Patients with higher scores on these scales showed lower LDAEP (Table 4). As shown in Table 3 and 4 all other psychopathological scales were not significant. LDAEP on both hemispheres were positively associated with medication by means of CPZ-equivalent dose (beta = 0.162, p = 0.001 and beta = 0.173, p = 0.030 for right and left hemisphere).

**Table 3:** Associations between right-hemispheric LDAEP values and clinical characteristics among patients.

Measures	B	95%-CI	Wald χ^2^ (df)	Sig
CPZ	0.162	0.069; 0.254	11.593 (1)	0.001*
PANSS positive	−0.014	−0.154; 0.125	0.041 (1)	0.840
PANSS negative	0.103	−0.036; 0.243	2.114 (1)	0.146
PANSS composite score	−0.086	−0.241; 0.070	1.170 (1)	0.279
PANSS general	−0.174	−0.354; 0.006	3.574 (1)	0.059
SANS Affect	0.207	0.094; 0.321	12.908 (1)	0.000*
SANS Alogia	−0.015	−0.151; 0.121	0.047 (1)	0.829
SANS Avolition	−0.101	−0.228; 0.026	2.416 (1)	0.120
SANS Anhedonia	0.155	0.029; 0.282	5.779 (1)	0.016*
SANS Attention	0.189	0.036; 0.341	5.906 (1)	0.015*
SANS composite score	0.153	0.011; 0.296	4.451 (1)	0.035*
BRMS	0.054	−0.129; 0.237	0.331 (1)	0.565
HAMD 17	−0.049	−0.204; 0.106	0.391 (1)	0.532
CDSS G	−0.055	−0.184; 0.074	0.695 (1)	0.404

Abbreviations: CPZ, Chlorpromazine Dose Equivalence Ratios; PANSS, Positive and Negative Syndrome Scale; SANS, Scale for Assessment of Negative Symptoms; BRMS, Bech-Rafaelsen Melancholia Scale; HAMD, Hamilton Depression Rating Scale, CDSS-G, Calgary Depression Rating Scale for Schizophrenia.

* p<0.05.

**Table 4:** Associations between left-hemispheric LDAEP values and clinical characteristics among patients.

Measures	B	95%-CI	Wald χ^2^ (df)	Sig
CPZ	0.173	0.160; 0.331	4.681 (1)	0.030*
PANSS positive	−0.091	−0.219; 0.037	1.935 (1)	0.164
PANSS negative	−0.061	−0.275; 0.152	0.320 (1)	0.572
PANSS composite score	−0.007	−0.237; 0.223	0.004 (1)	0.950
PANSS general	−0.159	−0.299; −0.019	4.962 (1)	0.026*
SANS Affect	−0.073	−0.256; 0.110	0.607 (1)	0.436
SANS Alogia	−0.048	−0.319; 0.224	0.118 (1)	0.731
SANS Avolition	−0.120	−0.329; 0.089	1.263 (1)	0.261
SANS Anhedonia	−0.219	−0.367; −0.071	8.406 (1)	0.004*
SANS Attention	−0.028	−0.276; 0.221	0.047 (1)	0.828
SANS composite score	−0.137	−0.294; 0.019	2.970 (1)	0.085
BRMS	−0.372	−0.493; −0.250	36.082 (1)	0.000*
HAMD 17	−0.075	−0.294; 0.143	0.457 (1)	0.499
CDSS G	−0.305	−0.409; −0.202	33.331 (1)	0.000*

Abbreviations: CPZ, Chlorpromazine Dose Equivalence Ratios; PANSS, Positive and Negative Syndrome Scale; SANS, Scale for Assessment of Negative Symptoms; BRMS, Bech-Rafaelsen Melancholia Scale; HAMD, Hamilton Depression Rating Scale, CDSS-G, Calgary Depression Rating Scale for Schizophrenia.

* p<0.05.

## Discussion

The present study was designed to investigate the role of serotonergic neurotransmission estimated by the LDAEP for the psychopathology of negative symptoms in schizophrenia. Due to the heterogeneity of the clinical concept of schizophrenia and its limitations as a valid object for scientific investigation [60], the level of psychopathological symptoms was chosen. We hypothesized that the LDAEP in patients with predominant negative symptoms would deviate from that of patients with predominant positive symptoms and healthy controls, indicating a difference in serotonergic neurotransmission. The results of this study provide new evidence in schizophrenia research. We would like to emphasize two remarkable findings. First, patients with schizophrenia showed a significantly stronger LDAEP than the control group. Based on the presumptions of the inverse relationship between LDAEP and 5-HT, this may indicate a difference in serotonergic neurotransmission. Moreover, the stronger LDAEP in patients with schizophrenia is highly associated with negative symptoms. Second, only the increased LDAEP in the right hemisphere was associated with negative symptoms, underscoring the effects of laterality in brain functions and brain activity in schizophrenia. The single electrode estimation at Cz did not show any significant differences between the groups, which may derive from additional frontal source activation involved in high intensities. This has also been reported by Hagenmuller et al. [58].

Our findings contrast with those of previous studies, which showed that patients with schizophrenia had a weaker LDAEP than healthy controls [37], [38], [39], [40]. However, those studies were not designed to control for LDAEP differences between positive and negative symptoms. They focused on schizophrenic patients as a self-contained group. Nevertheless, Juckel et al. [39] reported a tendency toward a positive relationship between PANSS negative score and LDAEP whereas Gudlowski et al. [37] found a negative relationship between those scores. Our findings are contrary to the results of Gudlowski et al. [37]. One explanation for inconsistent findings could be due to a difference in methodology as sampling biases, gender effects, intensity of stimuli and methods of estimation (DSA vs. single-electrode) [32]. In particular, our data were analysed with DSA method, whereas Gudlowski et al. [37] used single-electrode estimation for LDAEP. According to Hagenmuller et al. [58], studies using different methods are difficult if not impossible to compare. Furthermore, the sample in Gudlowski’s study included females and males. Even though some studies reported no gender effects [29], [40], [61], others have documented some effects on the LDAEP [62], [63], [64]. The study by Juckel et al. (2008) [39] used comparable methodology to the present study and some results are in line with our findings, showing strong LDAEP in patients with negative symptoms among schizophrenic patients. Compared to healthy controls, they reported weaker LDAEP in the left hemisphere in patients, which states a contrary result to our findings.

Nonetheless, our results are consistent with those of previous research on neurotransmitter alterations in negative symptoms and suggest the role of an impaired serotonergic system [12]. Although many studies on the direct involvement of the serotonin system in schizophrenia exist, here we focus on results concerning negative symptoms. Direct evidence is provided from a post-mortem study, which reported a decreased 5-HT2 receptor density in frontal cortex in patients with chronic schizophrenia [65]. Furthermore, a PET study showed lower availability of 5-HT1A receptors in patients with schizophrenia compared to healthy controls and receptor binding was negatively associated with negative symptoms, estimated by the PANSS scale [66]. Moreover, a genetic study by Reynolds [67] gives support to an involvement of the serotonergic system in the pathogenesis of negative symptoms, since the 5-HT2C receptor promoter polymorphism is associated with negative symptoms. From studies on the mechanism of action of atypical neuroleptics in negative symptoms an indirect evidence for serotonergic involvement is provided. In this context, it remains unclear why serotonin antagonists as well as agonists have an impact on the serotonergic system and improve the outcome of negative symptoms. Silver [20] suggests that these pharmacologically distinct treatments may share common final mechanism. This paradoxical finding needs further investigation. Moreover, one has to consider that different 5-HT receptors have opposite effects on the function of neurons by means of inhibition and excitation [15]. Further research is needed to clarify, if negative symptoms are caused directly by a primary abnormality in serotonergic transmission or in a secondary way via modulation of dopamine release [11], [68], [69].

With regard to the laterality effect, the present results showed a positive association between LDAEP and negative symptoms (SANS subscale affective flattening, anhedonia and attentional impairment, SANS composite score) in the right hemisphere, and a negative association between LDAEP and depressive symptoms (BRMS and CDSS G scale) in the left hemisphere in schizophrenic patients. A possible explanation could be that the LDAEPs in patients with high scores on depressive scales converge towards the LDAEP values of healthy controls (weaker LDAEP). This conclusion is in line with the literature about LDAEP in depressive patients, where no significant effect on the LDAEP has been shown [40], [63], [70]. Our results could also be due to serotonergic interhemispheric asymmetry, respectively to a reduced leftward asymmetry of brain structures of the auditory cortex in schizophrenia as observed by Salisbury et al. [71] and Shenton et al. [72]. At that juncture, that the role of the laterality effect in schizophrenia and in particular in negative symptoms is not known, interpretation is limited.

Nevertheless, our study is not without limitations: The sample size was relatively small, which had an effect on the statistical power. Furthermore, the effects of education were not considered. With regard to the influence of attention to the LDAEP [48] and given that patients with negative symptoms often show an attention deficit during auditory performances [73], an objective procedure controlling attention would have been necessary to add further consistency to our findings. A biased effect of medication is plausible since all patients were treated with atypical neuroleptics. There was an association with CPZ-equivalent dose and LDAEP found in both hemispheres in this study, indicating an elevated LDAEP (and lower serotonergic activity) with higher medication use. Furthermore, general symptoms rated on PANSS scale were negatively related to medication in that they displayed a statistical trend (p = 0.08). In a study by Juckel et al. [38] an increased LDAEP after a treatment with atypical neuroleptics compared to baseline was observed. Moreover, in a PET study, a trend toward a decreased 5-HT2 receptor binding in prefrontal cortex was found in neuroleptic treated patients, whereas neuroleptic naive patients showed similar results as healthy controls [74]. As negative symptoms also occur as pharmacological side effects (secondary negative symptoms) it is debatable if the found relationship between LDAEP and negative symptoms is an effect of secondary negative symptoms. A distinction between primary and secondary negative symptoms is not possible with contemporary measurements of psychopathology [75], [76]. On the other hand, a study design including unmedicated chronic schizophrenic patients is hardly realistic both for ethical reasons and practicability. Further studies with more focus on the effect of medication are therefore needed.

Another limitation is the possible influence of other neurotransmitters on the LDAEP. There are genetic association studies and challenge trials on possible influences of dopamine, glycine, and nitric oxide [33], [34], [35], [36]. As these studies point to a sensitivity of the LDAEP also to neurotransmitter systems other than 5-HT, the LDAEP’s specificity as a marker of serotonergic function is challenged [32]. This has to be taken into account in the interpretation of this study. Nevertheless, also these results are in part heterogeneous, e.g. an association of the LDAEP with the dopaminergic system by means of the *COMT* Val158Met-polymorphism [34] could not be reflected in a dopaminergic challenge trial [33].

In conclusion, the aim of the present study was to investigate the LDAEP as an indicator of serotonin functioning within the schizophrenic spectrum. In particular, we took account of the heterogeneity of clinical diagnosis by examining the accurate psychopathological symptoms. The results showed an association between the serotonergic function estimated by the LDAEP and the extent of negative symptoms directly. Our findings support the idea of differential clinical features of schizophrenia and contribute to the clarification of the aetiology of negative symptoms.

## Supporting Information

Figure S1 **Example of loudness dependence of auditory evoked potentials (LDAEP).** Auditory evoked activity of the tangential dipole in the right hemisphere following auditory stimulation of a 1000 Hz tone with different sound pressure levels (60 to 100 dB SPL) over all subjects (n = 26).
(EPS)

Figure S2 **Overall distribution of the loudness dependence of auditory evoked potentials (LDAEP) values between both groups.** The boxplots represent medians, quartiles and extreme values of the LDAEP variable in the left (A) and right (B) hemisphere across healthy controls and patients with schizophrenia.
(EPS)
